# A Study on Differential Effects of Mathematics Reading Ability on Students’ Value-Added Mathematics Achievements

**DOI:** 10.3390/bs13090754

**Published:** 2023-09-11

**Authors:** Cheng Zhu, Xiaopeng Wu

**Affiliations:** 1 School of Mathematics and Statistics, Qiannan Normal University for Nationalities, Duyun 558000, China; zhuchengmathedu@163.com; 2 Faculty of Education, Northeast Normal University, Changchun 130024, China

**Keywords:** mathematics reading ability, mathematics achievement, value-added assessment, education assessment

## Abstract

Value-added assessments have become a reasonable and accepted assessment method for education and teaching. Mathematics reading ability is an important ability in mathematics learning which provides a prerequisite for solving mathematical problems. With the aim of uncovering the effects of mathematics reading ability on the continuous development of mathematics learning, this study focuses on the value added to students’ mathematics reading ability as well as their mathematics performance. From a longitudinal perspective, we collected academic achievement data for 463 s-grade students, including their scores on their mathematics reading ability, which were then used a developed measurement tool. Building on Weiss’s “Theory of Change”, the students were divided into four categories: high academic achievement and high value-added, low academic achievement and high value-added, low academic achievement and low value-added, and high academic achievement and low value-added. Finally, we discussed the impact of the students’ reading abilities in mathematics on their overall achievement. This study reveals a close correlation between mathematics reading skills and value-added performance. Higher scores in mathematics reading indicate higher value-added levels. For students with initially high scores, their mathematics reading skills greatly contributed to their high value-added performance.

## 1. Introduction

Reading is the primary means by which human beings acquire information and knowledge, which is an integral part of their productive lives. In the international mathematics education community, the importance of mathematics reading has received widespread attention from researchers and educators. For example, the UK National Curriculum (Mathematics), implemented in September 2014, not only emphasized mathematical reasoning, mathematical language, and problem solving, but also integrated “reading and writing literacy” into the stage goals, which are Key Stage 1, Lower Key Stage 2, and Upper Key Stage 2 [1]. China’s mathematics curriculum standard for compulsory education (2011) [2] emphasized that diverse learning styles such as reading for self-study should be promoted, and teachers should guide students in reading for self-study.

In terms of the relationships between reading and academic achievement, some studies have explored the correlation between mathematics reading ability and mathematics achievement and found that these two share basic cognitive abilities to some extent, including memory, visualization, and language skills [3,4]. Most of these studies, however, have tended to explore their relationship among students with dyslexia [5,6]. Additionally, the educational assessment was outcome evaluation, which focuses only on test scores. This has been criticized for ignoring the students’ individual development and constraining teachers’ personalized teaching. The emerging value-added assessment makes up for the shortcomings of this result-oriented approach by focusing on the students’ longitudinal growth data and providing a basis for the students’ sustainable development, pursuing not only “attainment” but also “growth”. Therefore, building on the percentile rank residual model in the value-added assessment, this study analyzes longitudinal data based on students’ mathematical achievement and further investigates the relationship between students’ mathematical reading ability and their value-added mathematics achievements.

## 2. Literature Review

### 2.1. Mathematical Reading Ability and Achievements

According to existing research, scholars agree that reading is a complex cognitive psychological process that involves acquiring meaning from linguistic symbols. In order to acquire meaning, one needs to have the appropriate mental lexicon and be able to integrate the meaning of linguistic symbols [7].

Mathematics reading is different from general reading in that it has its own unique abstraction in terms of vocabulary and grammar. Mathematics reading is a psychological process of acquiring mathematical knowledge and skills, mathematical ideas, mathematical methods, mathematical abilities, and other mathematical achievements from mathematical materials through mental activities such as perceptual recognition, comprehension, memorization, evaluation, and the auxiliary participation of hypothesizing, proving, inducting, generalizing, judging, and reasoning. At the secondary school level, the content of mathematical reading includes the development processes of mathematical conclusions in mathematical textbooks, textbook examples, and test questions. In particular, when reading to understand the mathematical conclusions presented in textbooks, the core of reading at this time does not only lie in comprehension, but also in thinking in relation to the unfolding process of the conclusions that already existed in the past.

Young children develop reading and Mathematics skills at different rates. Some children’s numeracy skills develop rapidly until they are faced with word problems. This is because the verbal and formulaic information in mathematics can be difficult for children. When the learning task involves calculating sums, products, or quotients and the information is presented in numerical terms with the symbols ordered conventionally, students who understand algorithms can solve these problems and learn easily. When the learning task requires the student to decide whether to calculate the sum, product, or quotient and the information is hidden in sentences, the student needs to understand the language in the text before he or she can apply the appropriate algorithm. To solve these types of mathematics problems (which are a common part of school curricula), children need to learn to read mathematically [8].

Mathematics reading ability is an important factor affecting student learning in mathematics. Adams [9] stated that students read mathematics not only simply by reading words, but also by reading numbers and symbols for comprehension. However, students who have difficulty reading “mathematical language” are mostly weak in mathematics. The National Council of Teachers of Mathematics stated that to know mathematics is to do mathematics (1989). Alternatively, if students know mathematics, they know how to apply mathematics. Yu and Yang [10] have found that middle school students with mathematics dyslexia have more difficulties in learning. This difficulty can affect the students’ enthusiasm, self-confidence, and their mathematics achievements. Using 314 pairs of twins as their experimental sample, Hart et al. [11] found that the students’ performance in mathematics was not entirely determined by their general cognitive abilities. Their mathematics reading and problem-solving abilities, among others, were more significantly influenced by their genetics and environment. Vilenius-Tuohimaa et al. [12] found that mathematics reading and ability remained significantly correlated when controlling for two influential factors, gender and parental education. Vista [13] explored the role of students’ language background in their mathematics achievement growth and found that their language background did not affect the mediating effects of mathematical reading abilities between problem-solving skills and mathematics achievement growth. This study’s focus was on the role of reading skills in mediating the relationship between mathematical problem-solving skills and mathematics achievement, but it did not explore the value-added issue in mathematics achievement.

Based on the literature, it was easy to conclude that there exists a relationship between mathematics reading ability and mathematics achievement; however, limitations still exist. First, most previous research was cross-sectional, and there has been a lack of studies investigating the relationship between mathematics reading ability and mathematics achievement from a longitudinal perspective [14]. Moreover, most studies have explored the relationship between general reading and mathematics achievement in a broader sense, but not in any specific discipline. Additionally, most studies have focused only on words or vocabulary and not on comprehension in reading. Lastly, most research on reading has only studied students with dyslexia, which has limited the sample to a particular small group of participants [15,16].

### 2.2. Value-Added Assessment

In recent years, a new type of assessment called “value-added assessment” (VAA) has emerged in the field of educational assessment, with the aim of providing a scientific and objective evaluation of students’ academic progress. In the value-added assessment, a student’s achievement is not a single test score or an average of scores, but rather the amount of progress a student makes over a period [17]. The novelty of value-added evaluation is that it does not take into account initial student differences, instead using the degree to which the school is effective in helping students grow as a basis for evaluating school effectiveness and teacher effectiveness [18].

There have been some studies on teacher effectiveness since the introduction of value-added assessment. For instance, in one educational study of value-added assessment in Tennessee, Sanders et al. [19] found that students’ socioeconomic status did not have a significant effect on their achievement gains, and that teachers and schools had a far more dominant influence on the students than socioeconomic or family background factors [20,21]. This provided a reference for solving the problem of educational fairness and guided a new direction for educational research. Using this comprehensive model of school effectiveness, Jong et al. [22] empirically investigated Dutch schools. The study showed that in terms of school improvement, the time that teachers spent on students and the learning opportunities given to students were more effective for student achievement growth. For value-added student achievement, what needs to be considered is no longer the school choice, but rather more practical factors such as school internal management and teaching. In 2011, building on Sanders’s (1997) study, the Tennessee Department of Education (TDOE) developed the Tennessee Educator Acceleration Model (TEAM). TEAM conducts teacher effectiveness assessments based on combined information on student achievement and classroom observations. They found that the quality of teacher instruction in U.S. elementary and secondary schools still varied dramatically, even between classes in the same school [23], further affirming the importance of classroom instruction on student academic growth. During this same time, the Measurement of Effective Teaching (MET) project, supported by the Bill and Melinda Gates Foundation, used improvements in student achievement to estimate the value added by teachers. Specifically, based on classroom observations of teaching quality through the process dimensions, the MET project used improvements in student achievement as a measure of teacher quality. They found that teacher quality was unrelated to advanced degrees or certificates and instead more closely associated with the teachers’ experience [24].

Over the past half century, VAA has evolved in the direction of studying more specific objects, and research has shifted closer to teaching, learning, and student-focused investigations. VAA was initially used to explore educational equity at the national level, then to improve school effectiveness and teacher accountability. Gradually, it has moved to research on the student level, focusing on the impact of student factors on growth.

### 2.3. Model for Measuring Growth

There are seven commonly used growth measurement models: the gain score model, the trajectory model, the categorical model, the residual model, the student growth percentile (SGP) model, the projection model, and Tennessee Value-Added Assessment system [25]. This study used the residual model, which is one of the most popular models used at the state level in the United States to calculate and measure student growth. It is one of the most easily understood regression methods for continuous variables but not for dichotomous or ordinal variables. The basic calculation of the residual model involves creating a linear regression equation between the scores across two years. Specifically, based on the regression equation and the student’s score in the previous year, the student’s expected score is estimated for the current year, as well as the “residual” between the student’s expected score and the student’s actual score. If the actual score is higher than the expected score, the student has achieved satisfactory growth; otherwise, the growth is not satisfactory. In practice, the residuals are usually standardized by ranking the residuals in percentiles (PRR).

In terms of model generalizability, through simulations, Castellano and Ho [26] compared students’ growth percentiles and percentile rank residuals using known statistical distributions. They concluded that the percentile rank residuals and student growth percentile both worked well in terms of recovering conditional growth percentiles. In addition, the percentile rank residuals were found to work better for smaller sample sizes. Therefore, the residual model is more applicable to small-sample growth measures at the classroom level.

Previous studies have measured academic growth based on grade point averages or explored the correlation between single academic performance and mathematical ability. However, there is a relative lack of research on mathematical reading ability and value-added mathematical achievement. On the one hand, this could be due to the absence of a uniform scientific method that can be used to measure academic growth. On the other hand, it may be that research on value-added academic achievement requires long-term follow-up surveys and incurs research costs. However, from an educational perspective, growth within individual students deserves more attention [27,28,29].

Building on this literature review, this study explores the relationship between mathematical reading ability and value-added student achievement. Specifically, firstly, we track the students’ mathematics achievement in the second grade of an urban middle school in Guizhou Province for one semester. Secondly, we evaluated the students’ mathematical reading ability based on mathematical reading test papers. Finally, we explored the relationship between the students’ academic value-added and mathematics reading ability through the application of a unified value-added model.

## 3. Methods

### 3.1. Sample

In this study, students in their second year of junior high school in Duyun City, Guizhou Province were selected as the research participants. The main reason for selecting these students the research participants was that this school is a key middle school in the autonomous region and has comparatively high teacher and student qualities. The sample of this study was randomly selected in the second grade of junior high school, with 10 classes and 479 students. A total of 479 test papers were sent out, with 463 valid questionnaires returned (efficiency rate of 96.7%), 227 from boys and 236 from girls.

### 3.2. Achievement Measures

#### 3.2.1. Mathematics Achievement Measure

In a city in Guizhou province, students are given several mathematics exams each semester. The most representative ones are the midterm exams and the final exams at the end of each semester. Both exams are administered uniformly throughout the year, marked to a uniform standard, and scored using a percentage system. In value-added assessment, the data should be obtained from factual observations, but not experiments [30]. In this study, the source of the mathematics achievements was the two exam scores organized by the school in the first semester.

#### 3.2.2. Mathematics Reading Test

##### Test Paper Dimensional Division 

Based on the previous literature and Professor Yang’s definition of mathematical reading, this study considered mathematical reading as the active cognitive–psychological process of acquiring meaning from mathematical texts, of which the core elements are character encoding, linguistic conversions, and integrated comprehension, as well as three levels of mathematical reading comprehension [31].

Character encoding means that the reader needs to sift through the material to find useful information, discard irrelevant and distracting information, and process, arrange, and organize the input. Language conversions refer to the reader’s ability to cognize words, symbols, and graphics in reading materials and translate them into language. Integrated comprehension means that the readers can understand mathematical material, extract key information from the material, abstract the mathematical essence from the material, and logically reason to arrive at the correct conclusion.

##### Test Paper Development

With taking the teaching schedule into account, referring to the test papers, “Mathematical Reading Test Paper”, “Symbolic Language Influence on Mathematical Reading Test Paper”, and the dimension division provided above, four questions were obtained through modification (See Appendix A below).

The test paper consisted of one multiple-choice question and three short-answer questions, with five points for the multiple-choice question and ten, twelve, and thirteen points for the other three short-answer questions sequentially. The dimension of language conversion corresponded to questions 1 and 4 (1), the integrated comprehension corresponded to questions 2 and 3, and the character encoding corresponded to question 4 (2). Among them, question 1 examined graphic language, questions 2 and 4 examined textual language, and question 3 examined symbolic language. It is important to note that the kind of language tested was relative, as it involved language conversion. Therefore one question often involved several mathematical languages.

The efficacy of the test paper was initially tested in December 2022 in five second-year classes selected from two other secondary schools. The test was administered for 45 min, and the distribution and collection of the questionnaires was supervised by the teachers. A total of 239 test papers were distributed in the two schools, with a total of 224 valid test papers returned. The reliability of the test was moderate to high (*r* = 0.804), and the correlation analysis of the structural validity results is given in Table 1.

As can be seen from Table 1, each variable is significantly correlated with each other at the 0.01 level or the 0.05 level, indicating that the structure between the variables of the test paper was reasonable.

Based on the students’ responses to the questions, the difficulty of the test was calculated using the formula $P=\frac{\bar{x}}{X}$, where P is the difficulty coefficient of a question, $\bar{x}$ is the average score of the question, and X is the total score of the question. The larger the P value, the less difficult the question is; conversely, the smaller the P value, the more difficult the question is. The difficulty coefficient of the math reading test was 0.44, and the difficulty coefficients of the three questions were 0.1, 0.89, 0.36, and 0.33, sequentially. Therefore, the difficulty level of the test paper met the requirements. 

The formula for differentiation is $D=\frac{{\bar{x}}_{h}-{\bar{x}}_{l}}{X}$, where ${\bar{x}}_{h}$ represents the mean of the high group, ${\bar{x}}_{l}$ represents the mean of the low group, and X represents the total score of the question. Generally, a question with a discrimination of 0.4 or higher indicates that the question is well differentiated, a discrimination between 0.2–0.29 means that the question needs to be improved, and a score below 0.19 usually indicates that the question needs to be removed. It was found that the differentiation of each question in mathematics reading was 0.409, 0.336, 0.571, and 0.592. Overall, the test paper had a high degree of differentiation, indicating that the differentiation of the test paper questions was relatively good.

According to the preliminary test results, this test paper had good reliability and validity and could be officially administered.

### 3.3. Data Collection and Analysis

The data for this study were collected from two mathematics examinations in the second semester of 2022, the first semester in September 2022 and the second semester in January 2023, spanning a period of five months. We used SPSS 26.0 for Windows to remove the students’ missing data from the exams, calculate the means of each test score, and process the scores separately using the *z*-score method.

In this study, the percentage ranking residual method was chosen, which is a linear regression model that meets typical linear regression assumptions. These assumptions include the presence of a linear relationship between the dependent and independent variables, the absence of error in the measurement of the variables, and the independence of the errors with a normal distribution and homoscedasticity [32].

This type of growth model requires at least two tests. However, such models are not based on cross-grade longitudinal scales, and two or more tests do not require longitudinal scales, but rather linear and nonlinear statistical models such as regression models. These models use either the cohort norm group of students as a frame of reference or a large accumulation of historical and tracking data as a frame of reference to portray the growth obtained by students. The reasons for choosing residual model were as follows:

- The number of variables in the model is relatively low.
- The model yields data that are easy to understand.
- This study addresses the same group of students in the same school and does not need to consider out-of-school factors, as would be the case for covariate models.
- The model performs better for measuring student progress compared to more complex models [33].

The linear regression model, which is central to the calculation of the residuals of the percentile ranking [34], is formulated as follows:

$${\hat{Y}}_{ti}=\beta_{0}+\beta_{1}Y_{1i}+\ldots +\beta_{t-1}Y_{(t-1)i}+\varepsilon_{i}$$

where ${\hat{Y}}_{ti}$ denotes projected export achievement, $Y_{ti}$ denotes the academic performance of student i in year t, β is the regression coefficient, and ε is the residual.

Based on the students’ two-time academic performance in mathematics, we obtained the regression coefficients β and $\beta_{0}$, which were 1.446 and −36.243, respectively, to obtain the regression equation ${\hat{Y}}_{ti}=1.446Y_{(t-1)i}-36.243$. 

Based on the regression equation and a student’s entrance grade (midterm grade), the projected export achievement of the student can be calculated. Afterwards, the “residual” between the expected exit grade and the actual exit grade (final grade) is calculated, which represents the student’s longitudinal growth performance. The formula is as follows:

$$\varepsilon_{i}=Y_{ti}-{\hat{Y}}_{ti}$$

If the actual grade is higher than the expected grade (ε>0), the student has achieved more satisfactory growth. Otherwise, the growth is not satisfactory.

The percent position of student i is calculated based on how many students have residuals less than or equal to the residual of student i. It can be expressed algebraically as follows:

$$PRR=\frac{\#residuals\leq Y_{ti}-{\hat{Y}}_{ti}}{n}\times 100$$

This equation provides the percentile rank residual for the student, where $\#residuals\leq Y_{ti}-{\hat{Y}}_{ti}$ denotes the number of residuals less than or equal to student i, and n is the total number of students in the sample.

## 4. Results

### 4.1. Differences in Students’ Rankings in Value-Added and Outcome Evaluations

We ranked the 463 students in this study from highest to lowest based on their midterm grades, final grades, and value-added performance, with smaller numbers representing higher rankings. Comparing the two evaluation results with the absolute value of the difference in ranking position, the data showed that the maximum value of the difference in ranking position between the midterm and final exam results for the sample of 463 students was 321, the minimum value was 0, and the average value was 83.3067. The maximum value of the difference in ranking position between the value-added evaluation results and the midterm exam results was 461, the minimum value was 0, and the average value was 146.0475. We determined that in the value-added evaluation results considering the longitudinal performance, the students’ ranking position changed more. The overall ranking change mean was 62.7408, which was higher than the single outcome evaluation.

**Figure 1:**
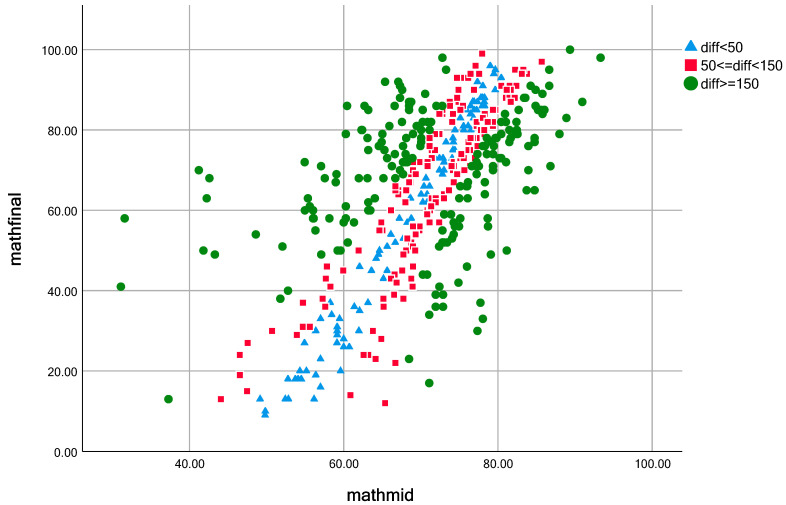
Bivariate scatterplot of midterm and final grades in mathematics.

To more visually demonstrate the differences between the two assessment results, Figure 1 supplements the summary statistics by identifying the types of students with midterm math grade rankings and value-added assessment result rankings. The absolute differences between the calculated midterm math grade rankings and the value-added assessment result rankings are coded based on size (diff < 50, 50 <= diff < 150, diff >= 150) and represented by different shapes displayed in bivariate scatter plots of students’ midterm and final math scores.

Based on the situation shown in Figure 1, students with an absolute difference in their place value greater than 150 are mostly distributed in the high and low score bands. The percentage of such students is 41.9% of the total. The students with an absolute difference in place values between 50 and 150, and students with an absolute difference within 50, are mostly distributed in the middle section of midterm grades, accounting for 36.1% and 22% of the total, respectively.

### 4.2. Differences in Mathematics Reading of Students at Different Value-Added Levels

#### 4.2.1. Overall Status of the Second-Year Students’ Mathematical Reading Ability

The scores of the math reading test paper were selected to indicate the students’ math reading ability, and the data were processed and counted according to the results of the students’ answers to the math reading test questions. The scores of the math reading test paper of all the students were combined and ranked, and the students in the top 27% of the sample size ranking were the high math reading group (125 students), the students in the bottom 27% of the sample size ranking were the low math reading group (125 students), and the students in the middle were the middle math reading group (213 students).

The mean score for the high math reading group was 26.0467, with a maximum value of 38 and a minimum value of 21; the mean score for the middle math reading group was 17.3562, with a maximum value of 21 and a minimum value of 13; and the mean score for the low math reading group was 10.0952. The overall mean score was 17.73. 

#### 4.2.2. Differences in Mathematics Reading among Students with Different High, Medium, and Low Value-Added Levels

The value-added scores (PRR) of each student in the second grade of middle school were combined in descending order, and the students in the top 27% of the sample size were selected as the high value-added level group, the students in the bottom 27% of the sample size were in the low value-added level group, and those in the middle were in the middle value-added level group. The mathematics reading scores of the three different value-added groups were calculated, and the average mathematics reading score of the high value-added group was 26, with a maximum value of 38 and a minimum value of 21; the average mathematics reading score of the medium value-added group was 17.3288, with a maximum value of 22 and a minimum value of 12; and the average mathematics reading score of the low value-added group was 10.1905. There were differences in the students’ reading abilities at different value-added levels. The results of the one-way ANOVA also showed that the F-value was 212.007 (*p* < 0.001), which indicated that there was a statistically significant difference in performance on the mathematics reading test among students with different value-added levels.

Table 2 of the multiple comparisons shows that the differences between the high value-added level, the medium value-added level, and the low value-added level were all significant. Specifically, the students at the high value-added level had higher mathematics reading ability than the students at the medium value-added level and low value-added level, and the difference between the high and low levels was significant. The students at the medium value-added level also had higher mathematics reading ability than the students at the low value-added level, and the difference was also significant. In other words, the higher the mathematics reading level, the higher the value-added level of mathematics academic achievement of the students, and the lower the mathematics reading level, the lower the value-added level of mathematics academic achievement of the students.

#### 4.2.3. Differences in Mathematics Reading between Male and Female Students

The math reading scores of boys and girls in the second grade of junior high school were counted separately, and the data were processed. The boys’ math reading scores had a minimum value of 5, a maximum value of 36, and a mean value of 17.6471, and the girls’ math reading scores had a minimum value of 5, a maximum value of 38, and a mean value of 17.8472. The maximum value of the girls’ math reading was higher than that of the boys, but the mean scores of both were very close. We conducted an independent samples t-test on the math reading scores of the boys and girls. 

The results of the independent samples *t*-test showed that the F-value was 0.179 (*p* = 0.673 > 0.05). Observing the *t*-value in the first row, the value of Sig was 0.850, which was greater than 0.05. Therefore, it can be concluded that there was no significant difference between the girls’ and boys’ math reading abilities.

### 4.3. Differences in Mathematics Reading in Initial Achievement and Mathematics Growth

When examining the value-added scores of the students’ performance in mathematics, we must acknowledge the fact that there was a relationship between academic growth and academic fundamentals. However, the two were not necessarily positively correlated. It is also common to encounter students with a low academic foundation but high growth, and students with a high academic foundation but low growth. Table 3 shows the results of the Pearson correlation between mathematics performance, PRR, and mathematics reading.

We can also see from Table 3 that the correlation between the math midterm scores and PRR (*r* = 0.037) was not significant, and there was a significant positive correlation between the rest of the variables. It also indicated, to some extent, that academic foundation did not significantly affect the value-added performance of the students.

In contrast, the growth achieved by such students was not recognized in many assessment approaches [35]. This was because in some schools, the students may achieve relatively good growth in achievement, but few of these students were able to pass the end-of-year proficiency exam (i.e., low status) because of their low initial achievement level. These students were in the schools that provided a high value-added education (relative to other schools), even though the student population, infrastructure, and faculty were not as robust. Similarly, in some schools, students may not exhibit significant growth or even experience regression, yet they all pass end-of-year proficiency exams (i.e., high status), but only because of the students’ high initial achievement levels. These students were in schools that did not provide a better value-added education, even though they were effective at maintaining student proficiency.

**Figure 2:**
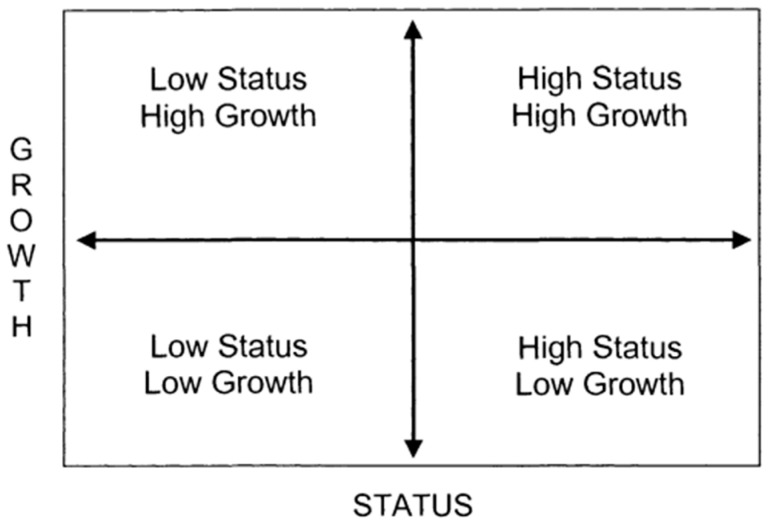
Status and growth.

Figure 2 is the “status and growth map” proposed by Weiss et al. (2008) [36], which provides a good illustration of how to consider the status and growth of student achievement. It divides the four areas based on a reference line according to “growth” and “status”, representing four different groups.

**Figure 3:**
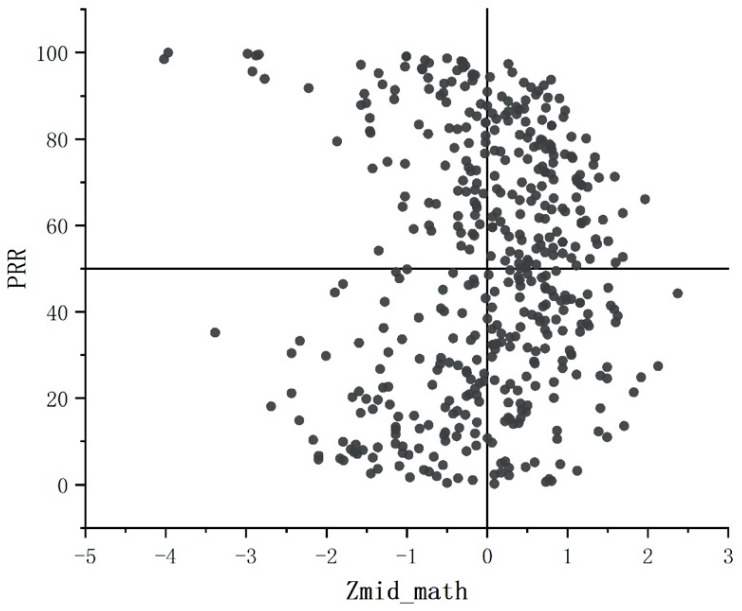
Progress and growth of students.

The relationship between the initial achievement and value-added measures in this study can be clearly observed with the aid of the “status and growth map”, which plots the base status of each student in this study on the *x*-axis and their “growth” on the *y*-axis, as shown in Figure 3.

In this map, the achievement base was the students’ midterm academic performance in mathematics (normalized). The “growth” was the percentage of residuals (PRR) calculated for each student based on the residual model. The two reference lines (PRR = 50 and zmid_math = 0) divided the coordinate system into four quadrants (the first quadrant in the upper right corner and the second, third, and fourth quadrants in counterclockwise rotation). The students in the first quadrant had greater than average midterm math scores (z > 0) and good value-added scores (PRR > 0). The students in the second quadrant had lower than average midterm scores (z < 0) in mathematics and had good value-added scores (PRR > 0). The students in the third quadrant had lower than average midterm scores (z < 0) in mathematics and did not have good value-added scores (PRR < 0), and students in the fourth quadrant had greater than average midterm scores (z > 0) in mathematics and did not have good value-added scores (PRR < 0).

The number of students located in each quadrant in Figure 3 is 138, 94, 111, and 120. The scatter density falling in the first quadrant was higher, which means that the students with a high academic foundation in mathematics had a greater chance of achieving high value-added scores. The scatter density in the second quadrant was the lowest, which means that the students with a poor academic foundation in mathematics had a lower chance of achieving high value-added scores. The mean values of PRR in the four quadrants were 70.6185, 81.5794, 20.7441, and 29.0461, respectively, and the value-added performance (PRR) of students with low initial scores in the second quadrant was closer to 100. Additionally, their mean PRR values were significantly higher than those in the first quadrant, which was also a high value-added area.

**Figure 4:**
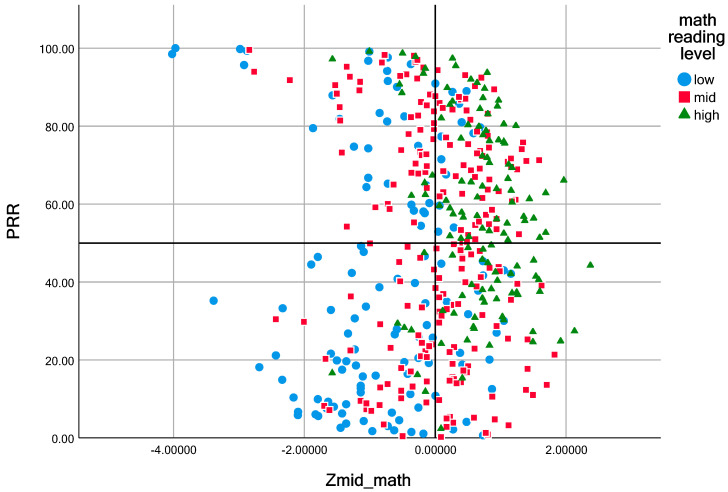
Student progress and growth chart with additional mathematics reading groupings.

To further explore the differences in mathematics reading levels across initial achievement and academic value-added scores, we coded the different reading levels with the symbols displayed in the images to facilitate a more visual observation of the relationship between the three measures of initial achievement, value-added level, and reading level, as shown in Figure 4.

In Figure 4, the overall reading levels were higher in the high-basic, high-value-added group and lower in the low-basic, low-value-added group. We analyzed the math reading scores in the four quadrants of the progress and growth chart to explore the differences in math reading levels based on initial achievement and academic value-added. The students in the first quadrant (high initial achievement and high value-added) had a mean reading score of 26.4928, with a maximum value of 38 and a minimum value of 12. The students in the second quadrant (low initial achievement and high value-added) had a mean reading score of 17.3638, with a maximum value of 38 and a minimum value of 5. The students in the third quadrant (low initial achievement and low value-added) had a mean reading score of 16.9468, with a maximum value of 36 and a minimum value of 5. Numerically, there was a difference in mathematical reading ability between the groups, with the students with a high initial achievement and high value-added score having a significantly higher mathematical reading ability than the other students. The results of the one-way ANOVA also showed that the F-value was 38.879 (*p* < 0.001), which indicated that there were significant differences in the performance of students with different value-added levels and initial scores in math reading.

Table 4 of the multiple comparisons shows that the difference between the strong fundamentals and high value-added group and the strong fundamentals and low value-added group was significant, and the difference between the rest of the groups was highly significant. Specifically, the students with high initial scores and high value-added scores had higher math reading scores than the students in all the other groups, and this difference between high and low scores was significant.

## 5. Discussion

Our study aimed to investigate the effect of mathematics reading on value-added mathematics achievement and concluded with two main findings. First, mathematics reading skills had a significant positive impact on value-added mathematics achievement, with higher mathematics reading scores associated with higher value-added levels. This positive result was consistent with previous research on similar mathematical reading and academic achievement, which involved conducting longitudinal studies on the relationship between early mathematical skills, reading comprehension, and mathematical problem-solving skills to explore the complex relationship between reading and mathematical abilities and illustrate the role of these abilities in promoting and influencing each other [37,38,39]. However, we found also a positive impact of mathematics reading on value-added mathematics achievement through a residual model from a value-added perspective. Although both contributed to mathematics achievement, the value-added levels differed from growth. Value-added refers to the extent to which a student’s actual academic achievement improves over a time period relative to the student’s own expected academic level. Achievement growth, on the other hand, refers to the current change in achievement relative to performance on a previous measurement or test. This suggests that in the future, more consideration could be given to these differences when improving the net effect of teachers’ influence on students’ academic achievement in mathematics, and that measures could be tailored to these characteristics. We also suggest further research into the mechanisms behind these differences and exploration of more relevant factors that influence the value added to academic achievement in mathematics.

Second, this study also found that mathematics reading ability was more conducive to high value-added performances for students with high initial scores than for students with low initial scores. According to Figure 4, “Progress and Growth”, the scatter density of high math reading level codes was higher in the high-basic high-value-added group than in the other groups in this study, and the difference in math reading scores across the four quadrants in Figure 4 also shows that the overall mean value of math reading was higher in the high-basic high-value-added group than in the high-basic low-value-added group with the same initial math scores. That is, more attention can be given to the top students in strengthening the instruction of mathematical reading skills instead of unilaterally taking the improvement of mathematical reading skills as a strategy to improve the mathematical performance of all students, which can accommodate the diversity of students and make teaching more efficient. Vigdor [40] and Kenan et al. [41] have also suggested that egalitarian shock improves the skills of underperforming students to some extent, but sharply dilutes the curriculum standards, negatively affecting top-performing students. Therefore, supporting differentiation by adapting the curriculum to meet the diverse instructional needs of students is the best way to promote higher achievement for all students. 

While mathematics reading does promote value-added performance, as seen in the exploration of differences in mathematics reading among students at different value-added levels, the difference between the mean values of mathematics reading performance in the low-base high-value-added group and the low-base low-value-added group was not significant. These values were close to or below the average overall, suggesting that there was indeed a positive relationship between mathematics reading and value-added scores. However, in the group of students with low initial achievement, this relationship does not seem to be significant. This may be explained by the floor effect: most studies have demonstrated that poor math reading skills may lead to a failure to improve math scores [42,43], and students at the lower end of the achievement scale may be dyslexic or lack math reading learning methods and strategies. For these students, the main factor affecting their performance improvement may not be math reading, but rather other mathematical skills [44,45]. This aspect warrants further investigation.

The results of this study should be understood in the context of several limitations. The residual model utilizes a common least squares (OLS) regression method, including the representation of OLS regression residuals as percentile ranks. Based on the characteristics of the model itself, which would describe growth in math achievement as linear, the reduction in growth due to the ceiling effect [46] cannot be captured here. Therefore, these results should be interpreted with caution, used reasonably for growth measurement, and avoided for teacher accountability [27]. However, this limitation did not prevent the selection of groups of students who achieved high value-added scores. When comparing the results of the two evaluations, we did see that there were some students with inverted achievement and growth, and we found groups of students who achieved high value-added scores (PRR > 50) despite poor academic performances. Additionally, using the residual model to calculate PRR does not require a large sample size [47], and it reduces experimental costs, simplifies the calculation process, and can be performed using general SPSS software or even EXCEL. Given these characteristics, it is a suitable growth prediction model [48]. Although this paper explored the value-added aspect of students’ academic achievement in mathematics through the lens of mathematics reading, the factors that affect the growth or value-added score of mathematics achievement cannot be exhausted, and the related theories still need to be explored further.

## Figures and Tables

**Table 1:** Results of structural validity tests of the mathematics reading test papers.

Variables	Character Encoding	Linguistic Conversions	Integrated Comprehension	Mathematics Reading
Character encoding	1	0.031 *	0.306 **	0.379 **
Linguistic conversions		1	0.136 **	0.400 **
Integrated comprehension			1	0.581 **
Mathematics reading				1

* *p* < 0.05; ** *p* < 0.01.

**Table 2:** Multiple comparisons of mathematical reading skills at different value-added levels.

(I) Value-Added Level	(J) Value-Added Level	Mean Difference (I-J)	SE	Sig.	95% Confidence Interval	
					Lower Bound	Higher Bound
High	Middle	8.67123 *	0.71858	0.000	6.9063	10.4362
	Low	15.80952 *	0.7366	0.000	14.0026	17.6165
Middle	High	−8.67123 *	0.71858	0.000	−10.4362	−6.9063
	Low	7.13829 *	0.46705	0.000	6.0034	8.2731
Low	High	−15.80952 *	0.73666	0.000	−17.6165	−14.0026
	Middle	−7.13829 *	0.46705	0.000	−8.2731	−6.0034

* The significance level for the difference in means is 0.05.

**Table 3:** Correlation between variables.

	Math (Mid)	Math (Final)	Mathematics Reading	PRR
Math (mid)	1			
Math (final)	0.664 **	1		
Mathematics reading	0.555 **	0.498 **	1	
PRR	0.037	0.749 **	0.178 **	1

** *p* < 0.01.

**Table 4:** Multiple comparisons of mathematics reading in the four value-added groups.

(I) Value-Added Group	(J) Value-Added Group	Mean Difference (I-J)	SE	Sig.	95% Confidence Interval	
					Lower Bound	Higher Bound
High status high value	Low status high value	3.92892 *	0.60351	0.000	2.7429	5.1149
	Low status low value	5.64591 *	0.57536	0.000	4.5152	6.7766
	High status low value	1.13442 *	0.56328	0.045	0.0275	2.2413
Low status high value	High status high value	−3.92892 *	0.60351	0.000	−5.1149	−2.7429
	Low status low value	1.71698 *	0.63255	0.007	0.4739	2.9600
	High status low value	−2.79450 *	0.62158	0.000	−4.0160	−1.5730
Low status low value	High status high value	−5.64591 *	0.57536	0.000	−6.7766	−4.5152
	Low status high value	−1.71698 *	0.63255	0.007	−2.9600	−0.4739
	High status low value	−4.51149 *	0.59429	0.000	−5.6794	−3.3436
High status low value	High status high value	−1.13442 *	0.56328	0.045	−2.2413	−0.0275
	Low status high value	2.79450 *	0.62158	0.000	1.5730	4.0160
	Low status low value	4.51149 *	0.59429	0.000	3.3436	5.6794

* The significance level for the difference in means is 0.05.

## Data Availability

The datasets generated during and/or analyzed in the current study are available from the corresponding author upon reasonable request.

## Appendix A

Mathematics Reading Test Papers
Class Name ID
1. On Sunday, Xiaotao walks from his home along a straight highway to a newsstand to read a newspaper, and after reading for some time, he returns home the same way. The function between the distance Xiaotao traveled from his home is *y* (*m*) and the time he spent *t* (min) is shown in the figure. The following statement is correct ( ). A. The distance from Xiaotao’s home to the newsstand is 900 m. B. The average speed of Xiaotao from his home to the newsstand is 60 m/min. C. The average speed of Xiaotao returning home from the newsstand is 80 m/min. D. Xiaotao spent 15 min reading the newspaper at the kiosk.
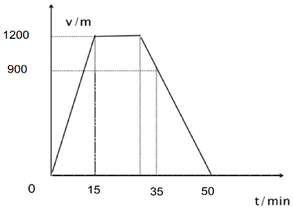

2. The definition of an equidistant sequence is: from the 2nd term onwards, the difference between each term and the previous term is the same constant, we call such a sequence an equidistant sequence, and we call this constant the tolerance of the equidistant sequence, which is usually represented by the letter d. For example: $a_{1}=3$, d=2, then $a_{2}=5,\;a_{3}=7,\;\ldots ,\;a_{n}=3+2(n-1)=2n+1$. An isoperimetric series is defined as a series in which the ratio of each term to the previous term from term 2 onwards is the same constant, we call such a series an isoperimetric series and call this constant the common ratio of the isoperimetric series, which is usually denoted by the letter q(q≠0). For example: $a_{1}=3,\;q=2,\;\mathrm{then}\;a_{2}=6,\;a_{3}=12,\;\ldots ,\;a_{n}=3\times 2^{\left(n-1\right)}$. (1) If the first term of an equidistant sequence $\left\{a_{n}\right\}$ is d and the common difference is $a_{n}=a_{1}+(n-1)d$, then the general formula for this equidistant series is *D*. Write the general formula for an equidistant series based on the representation of the general formula for an equidistant series, labelling the meaning of each letter in the formula. (2) The equal difference series has the following property: $a_{n}$, $a_{m}$ are any two terms in the equal difference series, and the relationship between $a_{n}$ and $a_{m}$ is $a_{n}=a_{m}+(n-m)d$. The proof process is as follows. $\begin{array}{l} \mathrm{left}=a_{1}+(n-1)d\\ \mathrm{right}=a_{1}+(m-1)d+(n-m)d=\mathrm{left} \end{array}$ Write the relationship between any two terms $a_{n}$ and $a_{m}$ in an isoperimetric series and justify your conclusion based on this property of the equidistant sequence.

3. Problem Background: In following figure, in isosceles triangle ΔABC, AB=AC, ∠BAC=120°, make AD⊥BC at point D, then D is the midpoint of BC, $∠BAD=\frac{1}{2}∠BAC=60{}^{\circ}$, so $\frac{BC}{AB}=\frac{2BD}{AB}=\sqrt{3}$. Application: As in following figure, ΔABC and ΔADE are isosceles triangles, ∠BAC=∠DAE=120°,D,E,C points *D*, *E* and *F* on the same line, connecting BD. (1) How many pairs of equal line segments are there in above figure and point out each of them. (2) Write an equation of equivalence between the line segments *CD*, *BD*, and *AD* in above figure. (Proof process required)
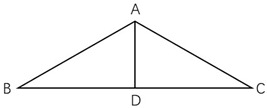
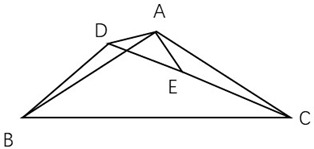

4. Read the following material before answering the questions that follow: In general, the multiplication of n identical numbers a, denoted $a^{n}$, such as $2^{3}=8$, when, 3 is called the logarithm with base 8 of 2, denoted $\log_{2}8$ (i.e., $3=\log_{2}8$). In general, if $a^{n}=b(a>0,a\neq 1,b>0)$ then n is called the logarithm of b with a as base, denoted $\log_{a}b$ (i.e., $\log_{a}b=n$). If $3^{4}=81$, 4 is called the logarithm of base 81 with 3, denoted $\log_{3}81=4$. (1) Calculate the value of each of the following logarithms: $\log_{2}4$ = _______; $\log_{2}16$ = _______; $\log_{2}64$ = _______. (2) Observe what relationship is satisfied between the three numbers 4, 16, and 64 in (1)? What relationship is satisfied between $\log_{2}4,\log_{2}16,\log_{2}64$? (3) Based on (2), can you write a general conclusion? $\log_{a}M+\log_{a}N=$______________(a>0, and a≠1, M>0, N>0)
